# Could remnant-like particle cholesterol become a risk factor in diabetic menopausal women with coronary artery disease? A cross-sectional study of single academic center in China

**DOI:** 10.1186/s12944-020-01224-8

**Published:** 2020-03-16

**Authors:** Xunxun Feng, Qianyun Guo, Shu Zhou, Tienan Sun, Yuyang Liu, Zhiming Zhou, Yujie Zhou

**Affiliations:** Department of Cardiology, Beijing Anzhen Hospital, Capital Medical University, Beijing Institute of Heart Lung and Blood Vessel Disease, Beijing Key Laboratory of Precision Medicine of Coronary Atherosclerotic Disease, Clinical center for coronary heart disease, Capital Medical University, Beijing, 100029 China

**Keywords:** Remnant-like particle cholesterol, Coronary artery disease, Menopausal women, Diabetes mellitus

## Abstract

**Background and aims:**

It has been confirmed that remnant-like particle cholesterol (RLP-C) mediates the progression of coronary artery disease (CAD). Currently there is limited information on RLP-C in menopausal women. With the special status of diabetes mellitus (DM) combined with the special body changes of the menopausal women, the RLP-C is particularly important when studying the changes that occurred in response to CAD and its associated risk factors. This study discussed whether RLP-C could be an independent risk factor for menopausal women with CAD and DM.

**Methods:**

The cohort consisted of 4753 menopausal women who had undergone coronary angiography. Subjects were separated into CAD and non-CAD groups, and univariate and multivariate logistic regression analysis of CAD risk factors were performed. All patients with a history of DM were divided into DM subgroups. Then, the univariate and multivariate logistic regression analysis of the risk factors of CAD and the comparison among age groups in the DM subgroup were performed. After age stratification of the DM group, the Kruskal-Wallis test was used to analyze the differences of various lipid indexes among age groups.

**Results:**

The multivariate logistic regression showed that RLP-C was an independent risk factor for CAD in menopausal women (OR 1.232, 95%CI 1.070–1.419). In the DM subgroup, it was also found that RLP-C was an independent risk factor for CAD (OR 1.366, 95%CI 1.043–1.791). Kruskal-Wallis test analysis found that RLP-C had no significant difference among three groups (*P* > 0.05).

**Conclusions:**

RLP-C was proved to be an independent risk factor for menopausal women with CAD and DM.

## Background

The high rates of morbidity and mortality of CAD are related to high levels of RLP-C [1]. Currently, the clinical lipid-lowering treatment of CAD patient’s targets low-density lipoprotein cholesterol (LDL-C), but among the many indexes of blood lipid, other potential targets remain. For CAD patients with decreased LDL-C, it is found that RLP-C may be an important target for the treatment of blood lipids in CAD patients after treatment of LDL-C [2]. RLP-C levels could also be used to predict the onset of CAD in different populations, thus confirming that RLP-C measurement is beneficial for risk assessment and treatment assessment of CAD patients [3]. Although RLP-C is a risk factor for cardiovascular related atherosclerotic disease, there is currently no standard method of measurement and the research potential of RLP-C is huge [4]. It has been found that an increase of 1 mmol/L in non-fasting RLP-C could increase the risk of ischemic heart disease by 2.8 times [5]. Studies have also confirmed that RLP-C affected the formation of atherosclerosis by affecting a variety of cells [6]. In addition, the RLP-C associates with atherosclerosis through blood lipid levels, insulin resistance, and it is frequently of abnormally high levels in diabetic patients [7]. While the importance of RLP-C on in-stent restenosis in DM patients has been confirmed, very few studies have been carried out in menopausal women with DM [8, 9]. Therefore, our aim was to determine whether RLP-C could be an independent risk factor in menopausal women with DM and CAD.

## Methods

### Study patients

Our study included 4753 menopausal women over the age of 50 who had undergone coronary angiography from January 2015 to December 2015. Subjects were designated into CAD (*n* = 2874) and non-CAD groups (*n* = 1897) according to coronary angiography assessments. Those with a history of DM were screened from the study population and divided into the DM subgroups, including DM combined with CAD group (*n* = 1031) and DM combined with non-CAD group (*n* = 465).

### Data collection

Demographic and clinical characteristics of patients were collected using standard case report forms, including age; body mass index (BMI); blood pressure including systolic blood pressure (SBP) and diastolic blood pressure (DBP); history of smoking, history of drinking; medical history including DM, hypertension (HT), hyperlipidemia, family history of cardiovascular disease (CVD); and medication therapy.

### Laboratory analysis

Fasting blood samples were obtained from study participants at the baseline. Total cholesterol (TC) and triglyceride (TG) levels were assessed in enzymatic assays. The levels of LDL-C and high-density lipoprotein cholesterol (HDL-C) were determined using homogeneous methods [10]. RLP-C calculation was defined as TC- (LDL-C + HDL-C) [11, 12].

### Statistics

Data were compared via the SPSS 22.0 statistical software package. Data were the mean ± SD for continuous variables, and ration for classified variables. Group differences were calculated in two-sided t-tests. Chi-square tests were used to represent classified variables which were shown as counts (percentage). Univariate analysis was carried out, and then these variables were selected and added to the multivariate analysis. Continuous variables across the age groups were compared using Kruskal-Wallis assessments. *P* < 0.05 (bilateral) was considered statistically significant.

## Results

### Baseline data

In the overall population, there were 1496 DM patients (1496/4753, 31.47%), accounting for more than 1/3 of the total population, which differed between the CAD and non-CAD groups (465 [24.75%] vs 1031 [35.87%]; *P* < 0.001). HT (3198/4753, 67.28%; 1191 [63.38%] vs 2007 [69.83%]; *P* < 0.001) and hyperlipidemia (1867/4753, 39.28%; 748 [39.81%] vs 1119 [38.94%]; *P* = 0.547) accounted for a very large proportion of the population and HT differed between CAD and non-CAD groups. This might be related to the high proportion of HT population in China and the characteristics of blood lipids in menopausal women. In terms of laboratory results of blood lipid, TG (1.63 ± 1.07 vs 1.75 ± 1.18; *P* < 0.001), HDL-C (1.18 ± 0.30 vs 1.10 ± 0.26; *P* < 0.001), and RLP-C (0.68 ± 0.43 vs 0.72 ± 0.47; *P* = 0.001) differed between the CAD and non-CAD groups (Table 1). In the laboratory results of the DM subgroup, TG (1.76 ± 1.03 vs 1.91 ± 1.45; *P* = 0.048), HDL-C (1.12 ± 0.29 vs 1.06 ± 0.25; *P* = 0.001), and RLP-C (0.70 ± 0.42 vs 0.76 ± 0.51; *P* = 0.024) differed across the CAD and non-CAD groups. No changes in LDL-C levels in either the general population or the DM subgroup (*P* > 0.05). (Table 2).

**Table 1:** Baseline of study population

	Total (*n* = 4753)	non-CAD (*n* = 1879)	CAD (*n* = 2874)	*P* value
Age, years	64.18 ± 7.42	63.27 ± 7.38	64.77 ± 7.39	<0.001
BMI, kg/m^2^	25.15 ± 3.29	24.98 ± 3.07	25.27 ± 3.43	<0.001
SBP, mmHg	129.08 ± 15.70	127.63 ± 15.74	130.03 ± 15.60	<0.001
DBP, mmHg	74.98 ± 11.83	73.88 ± 11.26	75.70 ± 12.14	<0.001
Smoking, n(%)	309 (6.50)	102 (5.43)	207 (7.20)	0.015
Drinking, n(%)	14 (0.29)	6 (0.32)	8 (0.28)	0.799
Medical history, n(%)				
DM, n (%)	1496 (31.47)	465 (24.75)	1031 (35.87)	<0.001
HT, n (%)	3198 (67.28)	1191 (63.38)	2007 (69.83)	<0.001
Hyperlipidemia, n(%)	1867 (39.28)	748 (39.81)	1119 (38.94)	0.547
Family history of CVD, n(%)	242 (5.09)	115 (6.12)	127 (4.42)	0.009
Laboratory results				
TC, mmol/L	4.46 ± 1.08	4.48 ± 1.04	4.45 ± 1.11	0.348
TG, mmol/L	1.70 ± 1.14	1.63 ± 1.07	1.75 ± 1.18	<0.001
LDL-C, mmol/L	2.63 ± 0.90	2.63 ± 0.86	2.62 ± 0.92	0.957
HDL-C, mmol/L	1.13 ± 0.28	1.18 ± 0.30	1.10 ± 0.26	<0.001
non-HDL-C, mmol/L	3.33 ± 1.04	3.30 ± 0.99	3.35 ± 1.07	0.141
RLP-C, mmol/L	0.71 ± 0.45	0.68 ± 0.43	0.72 ± 0.47	0.001
Medical treatment, n(%)				
Aspirin	4109 (86.45)	1541 (82.01)	2568 (89.35)	<0.001
Clopidogrel	2933 (61.71)	812 (43.21)	2121 (73.80)	<0.001
Statins	3476 (73.13)	1360 (72.38)	2116 (73.63)	0.343
β-blockers	2941 (61.88)	1092 (58.12)	1849 (64.34)	<0.001
ARB	1005 (21.14)	369 (19.64)	636 (22.13)	0.040
ACEI	242 (5.09)	74 (3.94)	168 (5.85)	0.003

*BMI* body mass index, *SBP* systolic blood pressure, *DBP* diastolic blood pressure, *DM* diabetes mellitus, *HT* hypertension, *CVD* cardiovascular disease, *CAD* coronary artery disease, *TC* total cholesterol, *TG* triglyceride, *LDL-C* low-density lipoprotein cholesterol, *HDL-C* high-density lipoprotein cholesterol, *RLP-C* remnant-like particle cholesterol, *ACEI* angiotensin converting enzyme inhibitor, *ARB* angiotensin receptor blocker

**Table 2:** Baseline of DM population

	DM Total (*n* = 1496)	non-CAD (*n* = 465)	CAD (*n* = 1031)	*P* value
Age, years	65.02 ± 7.17	64.70 ± 7.35	65.16 ± 7.09	0.252
BMI, kg/m^2^	25.34 ± 3.39	25.24 ± 3.10	25.39 ± 3.52	0.433
SBP, mmHg	129.16 ± 15.47	127.35 ± 14.81	129.97 ± 15.70	0.002
DBP, mmHg	75.19 ± 11.44	73.95 ± 11.36	75.74 ± 11.43	0.005
Smoking, n(%)	85 (5.68)	21 (4.52)	64 (6.21)	0.191
Drinking, n(%)	2 (0.13)	0 (0.00)	2 (0.19)	0.342
Medical history, n(%)				
HT, n (%)	1176 (78.61)	363 (78.06)	813 (78.86)	0.730
Hyperlipidemia, n(%)	606 (40.51)	190 (40.86)	416 (40.35)	0.852
Family history of CVD, n(%)	70 (4.68)	23 (4.95)	47 (4.56)	0.743
Laboratory results				
TC, mmol/L	4.36 ± 1.09	4.34 ± 1.05	4.37 ± 1.11	0.708
TG, mmol/L	1.86 ± 1.34	1.76 ± 1.03	1.91 ± 1.45	0.048
LDL-C, mmol/L	2.54 ± 0.87	2.53 ± 0.84	2.54 ± 0.89	0.761
HDL-C, mmol/L	1.08 ± 0.26	1.12 ± 0.29	1.06 ± 0.25	0.001
non-HDL-C, mmol/L	3.28 ± 1.04	3.23 ± 1.02	3.31 ± 1.05	0.195
RLP-C, mmol/L	0.74 ± 0.48	0.70 ± 0.42	0.76 ± 0.51	0.024
Medical treatment, n(%)				
Insulin	202 (13.50)	54 (11.61)	148 (14.35)	0.151
Aspirin	1315 (87.90)	390 (83.87)	925 (89.72)	0.001
Clopidogrel	981 (65.57)	212 (45.60)	769 (74.59)	<0.001
Statins	1113 (74.40)	344 (73.98)	769 (74.59)	0.803
β-blockers	938 (62.70)	289 (62.15)	649 (62.95)	0.768
ARB	306 (20.45)	97 (20.86)	209 (20.27)	0.794
ACEI	73 (4.88)	18 (3.87)	55 (5.33)	0.224

*BMI* body mass index, *SBP* systolic blood pressure, *DBP* diastolic blood pressure, *HT* hypertension, *CVD* cardiovascular disease, *CAD* coronary artery disease, *TC* total cholesterol, *TG* triglyceride, *LDL-C* low-density lipoprotein cholesterol, *HDL-C* high-density lipoprotein cholesterol, *RLP-C* remnant-like particle cholesterol, *ACEI* angiotensin converting enzyme inhibitor, *ARB* angiotensin receptor blocker

### CAD risk factor assessment through univariate model assessments

**Fig. 1:**
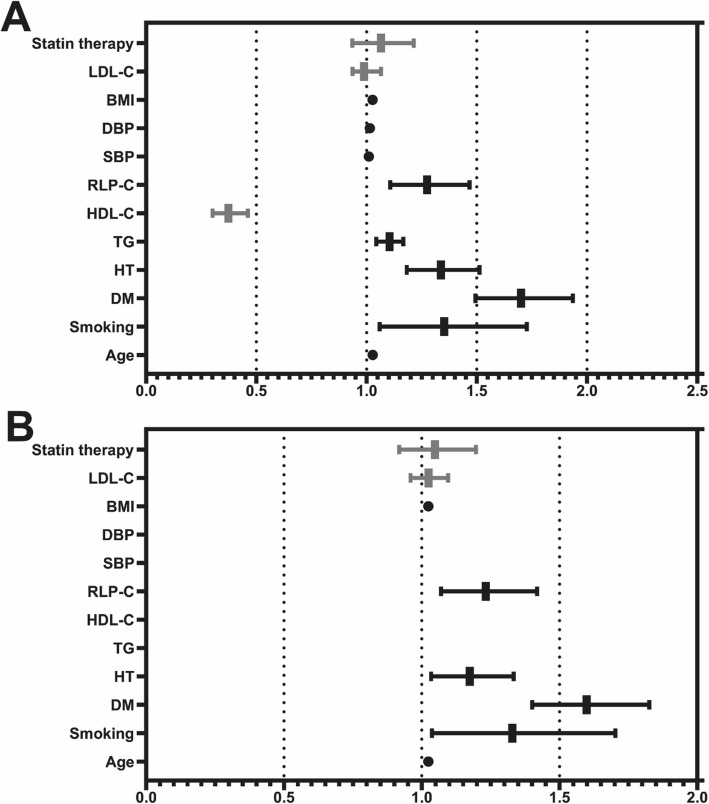
Univariate and multivariate logistic regression analysis of total study population. **a**: the univariate logistic regression analysis in total study population (Statin therapy: OR 1.065, 95%CI 0.935–1.214, *P* = 0.343; LDL-C: OR 0.988, 95%CI 0.924–1.029, *P* = 0.355; BMI: OR 1.027, 95%CI 1.009–1.046, *P* = 0.003; DBP: OR 1.015, 95%CI 1.009–1.020, *P* < 0.001; SBP: OR 1.010, 95%CI 1.006–1.014, *P* < 0.001; RLP-C: OR 1.274, 95%CI 1.107–1.467, *P* = 0.001; HDL-C: OR 0.373, 95%CI 0.301–0.462, *P* < 0.001; TG: OR 1.104, 95%CI 1.044–1.166, *P* < 0.001; HT: OR 1.337, 95%CI 1.182–1.512, *P* < 0.001; DM: OR 1.701, 95%CI 1.494–1.936, *P* < 0.001; Smoking: OR 1.352, 95%CI 1.059–1.727, *P* = 0.016; Age: OR 1.028, 95%CI 1.020–1.036, *P* < 0.001). **b**: the multivariate logistic regression analysis with model of Statin therapy, LDL-C, BMI, RLP-C, HT, DM, Smoking and Age (Statin therapy: OR 1.048, 95%CI 0.918–1.197, *P* = 0.488; LDL-C: OR 1.025, 95%CI 0.959–1.096, *P* = 0.462; BMI: OR 1.024, 95%CI 1.006–1.042, *P* = 0.011; RLP-C: OR 1.232, 95%CI 1.070–1.419, *P* = 0.004; HT: OR 1.174, 95%CI 1.034–1.334, *P* = 0.014; DM: OR 1.599, 95%CI 1.401–1.826, *P* < 0.001; Smoking: OR 1.329, 95%CI 1.037–1.703, *P* = 0.025; Age: OR 1.024, 95%CI 1.016–1.032, *P* < 0.001). LDL-C low-density lipoprotein cholesterol, BMI body mass index, DBP diastolic blood pressure, SBP systolic blood pressure, RLP-C remnant-like particle cholesterol, HDL-C high-density lipoprotein cholesterol, TG triglyceride, HT hypertension, DM diabetes mellitus

In the overall population, univariate analysis showed that age (OR 1.028, 95%CI 1.020–1.036, *P* < 0.001), BMI (OR 1.027, 95%CI 1.009–1.046, *P* = 0.003), SBP (OR 1.010, 95%CI 1.006–1.014, *P* < 0.001), DBP (OR 1.015, 95%CI 1.009–1.020, *P* < 0.001), family history of CVD (OR 0.709, 95%CI 0.547–0.919, *P* = 0.009), HDL-C (OR 0.373, 95%CI 0.301–0.462, *P* < 0.001), HT (OR 1.337, 95%CI 1.182–1.512, *P* < 0.001), DM (OR 1.701, 95%CI 1.494–1.936, *P* < 0.001), TG (OR 1.104, 95%CI 1.044–1.166, *P* < 0.001), RLP-C (OR 1.274, 95%CI 1.107–1.467, *P* = 0.001) were risks of CAD with obvious and positive effects, while statin therapy, drinking history, hyperlipidemia, TC, LDL-C, and non-HDL-C were not significant risk factors for CAD (*P* > 0.05). In the DM subgroup population, HDL-C (OR 0.478, 95%CI 0.317–0.719, *P* < 0.001) and RLP-C (OR 1.354, 95%CI 1.037–1.767, *P* = 0.026) were significant risks for CAD. (Fig. 1).

### CAD risk factors through multivariate logistic regression

**Fig. 2:**
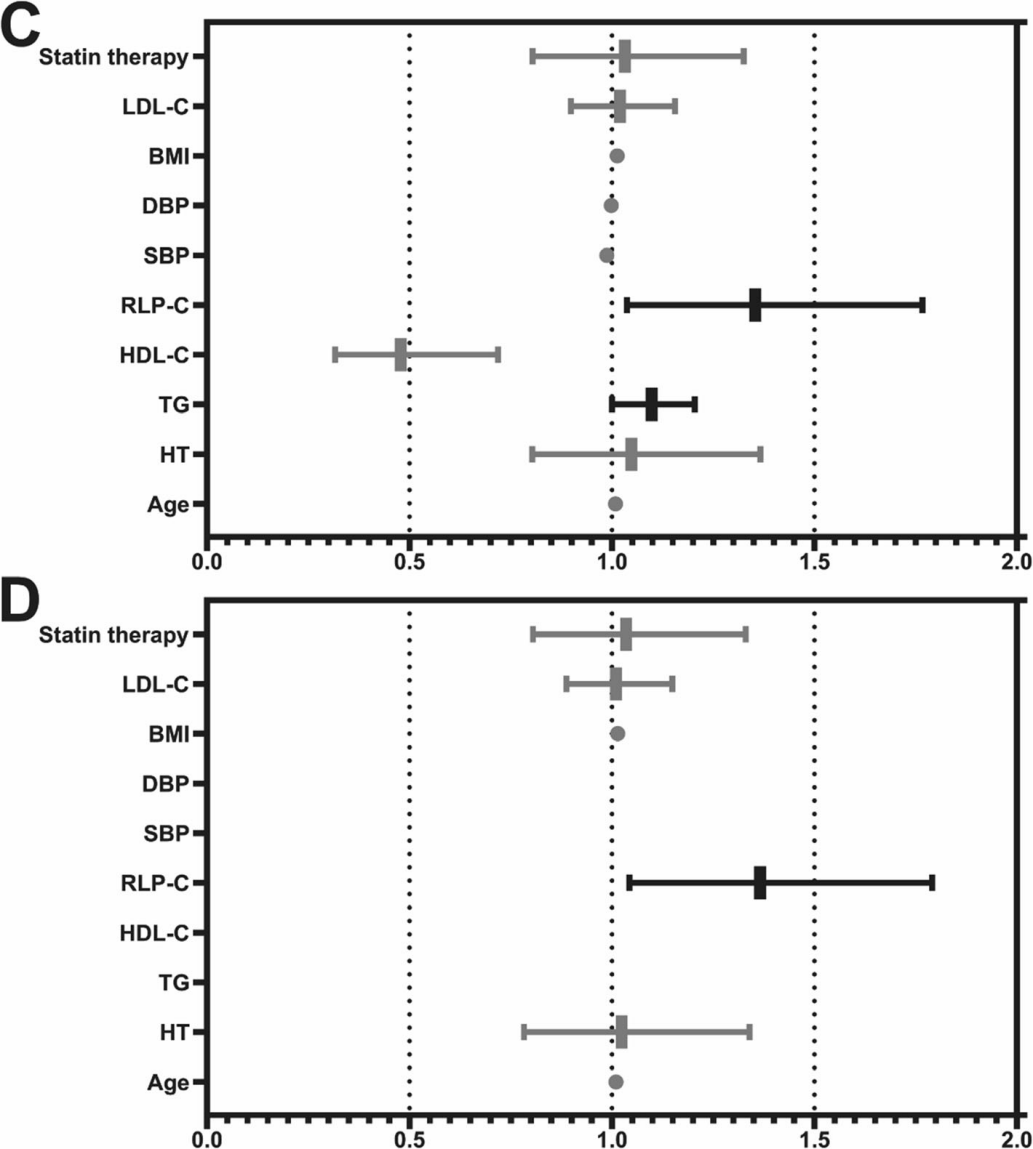
Univariate and multivariate logistic regression analysis of DM population. C: the univariate logistic regression analysis in DM population (Statin therapy: OR 1.032, 95%CI 0.804–1.326, *P* = 0.803; LDL-C: OR 1.020, 95%CI 0.899–1.156, *P* = 0.761; BMI: OR 1.013, 95%CI 0.981–1.046, *P* = 0.433; DBP: OR 0.998, 95%CI 0.990–1.007, *P* = 0.704; SBP: OR 0.988, 95%CI 0.981–0.994, *P* < 0.001; RLP-C: OR 1.354, 95%CI 1.037–1.767, *P* = 0.026; HDL-C: OR 0.478, 95%CI 0.317–0.719, *P* < 0.001; TG: OR 1.098, 95%CI 1.000–1.205, *P* = 0.050; HT: OR 1.048, 95%CI 0.803–1.367, *P* = 0.730; Age: OR 1.009, 95%CI 0.994–1.025, *P* = 0.252). D: the multivariate logistic regression analysis with model of Statin therapy, LDL-C, BMI, RLP-C, HT and Age (Statin therapy: OR 1.035, 95%CI 0.805–1.331, *P* = 0.789; LDL-C: OR 1.010, 95%CI 0.888–1.149, *P* = 0.879; BMI: OR 1.014, 95%CI 0.982–1.048, *P* = 0.391; RLP-C: OR 1.366, 95%CI 1.043–1.791, *P* = 0.024; HT: OR 1.024, 95%CI 0.783–1.340, *P* = 0.863; Age: OR 1.010, 95%CI 0.994–1.026, *P* = 0.214). LDL-C low-density lipoprotein cholesterol, BMI body mass index, DBP diastolic blood pressure, SBP systolic blood pressure, RLP-C remnant-like particle cholesterol, HDL-C high-density lipoprotein cholesterol, TG triglyceride, HT hypertension

In the overall population, after adjusting for age, BMI, smoking history, statin therapy, DM, HT, and LDL-C and RLP-C, regression analysis in overall population revealed RLP-C would be a strong CAD risk (OR 1.232, 95%CI 1.070–1.419, *P* = 0.004). It was also noted that RLP-C could be a significant risk of CAD in the DM population (OR 1.366, 95%CI 1.043–1.791, *P* = 0.024). (Fig. 2).

### Kruskal-Wallis test analysis

The DM subgroup was grouped according to age and divided into three groups: women aged 50–59, 60–69, or ≥ 70 years old. It was found that in different populations, blood lipid indicators included LDL-C, TG, TC, non -HDL-C differed across the groups of those ≥70 years old, which were higher than the other assessed groups (LDL-C, *P* = 0.001; TG, *P* = 0.003; TC, *P* < 0.001; non-HDL-C, *P* < 0.001), while RLP-C (*P* = 0.191) and HDL-C (*P* = 0.524) showed no changes across the three groups. (Tables 3,4).

**Table 3:** Age stratification of DM population

	Total (*n* = 1496)	non-CAD (*n* = 465)	CAD (*n* = 1031)	*P* value
TC, mmol/L				
50–59	4.43 ± 1.12	4.44 ± 1.14	4.43 ± 1.11	0.935
60–69	4.41 ± 1.11	4.42 ± 1.06	4.41 ± 1.11	0.918
≥ 70	4.16 ± 0.98	4.06 ± 0.86	4.21 ± 1.03	0.178
TG, mmol/L				
50–59	1.97 ± 1.49	1.77 ± 1.01	2.08 ± 1.69	0.032
60–69	1.87 ± 1.26	1.83 ± 1.06	1.88 ± 1.34	0.649
≥ 70	1.74 ± 1.33	1.58 ± 0.96	1.81 ± 1.46	0.085
LDL-C, mmol/L				
50–59	2.59 ± 0.87	2.60 ± 0.87	2.58 ± 0.87	0.909
60–69	2.59 ± 0.90	2.58 ± 0.87	2.59 ± 0.91	0.880
≥ 70	2.36 ± 0.79	2.32 ± 0.73	2.38 ± 0.82	0.498
HDL-C, mmol/L				
50–59	1.07 ± 0.26	1.12 ± 0.28	1.04 ± 0.24	0.004
60–69	1.08 ± 0.27	1.12 ± 0.30	1.06 ± 0.26	0.007
≥ 70	1.09 ± 0.25	1.10 ± 0.28	1.08 ± 0.24	0.692
non-HDL-C, mmol/L				
50–59	3.36 ± 1.06	3.31 ± 1.08	3.38 ± 1.05	0.539
60–69	3.34 ± 1.06	3.30 ± 1.05	3.35 ± 1.07	0.563
≥ 70	3.08 ± 0.96	2.97 ± 0.81	3.13 ± 1.01	0.128
RLP-C, mmol/L				
50–59	0.77 ± 0.56	0.71 ± 0.54	0.79 ± 0.56	0.176
60–69	0.75 ± 0.46	0.72 ± 0.39	0.76 ± 0.48	0.294
≥ 70	0.72 ± 0.46	0.65 ± 0.31	0.75 ± 0.51	0.035

*TC* total cholesterol, *TG* triglyceride, *LDL-C* low-density lipoprotein cholesterol, *HDL-C* high-density lipoprotein cholesterol, *RLP-C* remnant-like particle cholesterol

**Table 4:** Kruskal-Wallis test between three age groups

	*P* value					
	TC	TG	LDL-C	HDL-C	non-HDL-C	RLP-C
50–59 vs 60–69	1.000	1.000	1.000	0.524	1.000	0.191
≥70 vs 50–59	0.005	0.007	0.001		0.001	
≥70 vs 60–69	0.003	0.009	<0.001		0.001	

*TC* total cholesterol, *TG* triglyceride, *LDL-C* low-density lipoprotein cholesterol, *HDL-C* high-density lipoprotein cholesterol, *RLP-C* remnant-like particle cholesterol

## Discussion

### Main findings

We found that in the overall population of menopausal women, the RLP-C differed in CAD compared to non-CAD groups (0.68 ± 0.43 vs 0.72 ± 0.47, *P* = 0.001). When the DM subgroup population was further investigated, the RLP-C differed between CAD and non-CAD menopausal women (0.70 ± 0.42 vs 0.76 ± 0.51, *P* = 0.024). The RLP-C showed no change according to age in diabetic women (TC, P = 0.001; TG, *P* = 0.003; LDL-C, *P* < 0.001; HDL-C, *P* = 0.524; RLP-C, *P* = 0.191), confirming that along with the age growing, RLP-C might be more stable than other kinds of lipids among the menopausal women with CAD and DM.

### Inspiration from basic and clinical studies of dyslipidemia in menopausal women with DM and CAD

### LDL-C and TG

The blood lipid in DM patients was characterized by the decreased HDL-C levels, the increased TG-rich lipoprotein levels, and abnormal composition of high-density lipoprotein (HDL), low-density lipoprotein (LDL), and TG-rich lipoprotein particles [13]. The 2019 European Society of Cardiology guidelines recommended that for the high-risk type 2 DM patients, the LDL-C levels should be reduced to more than 50% of the baseline levels, and target levels of LDL-C were ≤ 1.8 mmol/L [14]. However, LDL-C in the overall population and subgroups of female patients failed to reach the ascribed standards. It was also seen that the LDL-C levels noted in this study were not consistent with common CAD and non-CAD populations, which was generally around 2.6 mmol/L (overall 2.63 ± 0.86 vs 2.62 ± 0.92, *P* = 0.957; subgroup 2.53 ± 0.84 vs 2.54 ± 0.89, *P* = 0.761). In addition, even at low concentrations of LDL-C controlled by statins, RLP-C could still be used as a risk factor for CAD [15]. In our study population, we found that the predictive effect of LDL-C on CAD might be less than RLP-C. In addition, studies had shown that the risk of patients with CAD and DM was related to TG metabolic disorders [16]. Previous studies had found that while elevated TG levels in DM patients did not represent an independent marker of cardiovascular events, high serum TG was associated with CVD [17]. We also found that in menopausal women, the TG levels of the overall population and DM subgroup population in the CAD group significantly differed to the non-CAD group (overall group [1.63 ± 1.07 vs 1.75 ± 1.18], *P* < 0.0001; subgroup [1.76 ± 1.03 vs 1.91 ± 1.45], *P* = 0.048). RLP-C was a known risk factor for CVD in women and provides more information than TG [18]. Furthermore, TG and HDL-rich lipoproteins subgroups, such as remnant-like particle (RLP), could better predict the occurrence of CAD than TG and HDL [19].

### RLP-C

Studies had found that an increase of 1 mmol/L in non-fasting RLP-C could increase the risk of ischemic heart disease by 2.8 times, but was not related to the decrease of HDL-C [5]. Compared to HDL-C, LDL-C and other lipids, RLP-C, which was mainly composed of very-low-density lipoprotein cholesterol (VLDL-C) and intermediate-density lipoprotein cholesterol (IDL-C), was not a common clinical lipid measurement indicator. Through direct measurement, it had been found that 1/3 of total cholesterol in plasma exists in RLP-C, that was in the TG-rich intermediate-density lipoprotein (IDL) and very-low-density lipoprotein (VLDL) [20]. Based on the fact that RLP-C was independent of LDL-C and was related to some extent with the body’s inflammatory reaction [21, 22], and the increase of RLP-C was related to low degree inflammation, combined with many studies, it was pointed out that RLP-C had an important relationship with CAD [23]. At the same time, studies found that the menopausal women with CAD were more likely to develop DM than those without CAD. It was seen that RLP-C levels in women with DM were higher than that of women without DM [24]. Interestingly, triglyceride-rich lipoproteins (VLDL+IDL) might be associated with carotid atherosclerosis among menopausal women [25]. Therefore, increased RLP-C was an important risk factor for CAD that can predict future coronary events in DM and CAD patients [26]. We found that the RLP-C level was significantly higher in the menopausal women with CAD and the DM subgroup than that in the non-CAD group (overall group [0.68 ± 0.43 vs 0.72 ± 0.47], *P* = 0.001; subgroup [0.70 ± 0.42 vs 0.76 ± 0.51], *P* < 0.024). Further, especially in the subgroup, the multivariate analysis showed that RLP-C was an independent risk factor for CAD while other traditional CAD risks might not be significantly positive.

Studies had found that RLP-C affected the formation of atherosclerosis by affecting a variety of cells [6]. The expression of low-density lipoprotein receptor-1 (LOX-1) was found to be enhanced in endothelial cells at high glucose concentrations in patients with CAD and DM [27]. Interestingly, RLP-C could also increase the expression of LOX-1 receptor protein by inducing the formation of superoxide associated with deoxyribonucleic acid breakage in endothelial cells, which could cause damage to endothelial cells and aggravate atherosclerosis [28]. Moreover, especially in the case of postprandial hyperlipidemia, DM and metabolic syndrome, the LOX-1 mediated RLP-C uptake mediated atherosclerosis by inducing the expression of LOX-1 [29]. At the same time, some studies had found that the level of LOX-1 was higher in obese menopausal women, which might be related to the increase of LOX-1 expression in adipose tissue [30]. We also speculated that in menopausal women with DM and CAD, RLP-C might also affect the pathogenesis of CAD by increasing the expression of LOX-1. Further research was required to determine the specific mechanism of action.

### Changes of RLP-C in special body conditions of menopausal women with DM and CAD

DM was a risk factor for CAD, and the study of RLP-C in DM had shown that RLP-C was closely related to the internal stenosis of stent in DM patients [10]. From the perspective of gender, menopausal women with DM showed serious metabolic problems, therefore atherosclerosis was more likely to occur in this population [31]. Long-term studies had found that female patients had a lower risk of CVD at a young stage due to the protective effect of estrogen [32]. Due to the loss of estrogen protection in menopausal women with CAD and various metabolic reasons, especially the negative metabolic effects caused by DM and obesity [33, 34], the incidence of CAD in menopausal women with DM had increased significantly. Epidemiological studies had found that the mortality rate of CVD associated with early menopause was high, but the relationship between menopausal age and postmenopausal CAD was unclear [35]. Some studies had also pointed out that there was a lack of prospective studies of the relationship between acute coronary syndrome (ACS) and menopausal age in menopausal women, and the prognosis of late menopausal women after ACS was better than that of early menopausal women [36]. Therefore, we further explored the changes of RLP-C in different age groups of DM patients. Compared with other lipids, the RLP-C was not significantly different among different age groups (*P* = 0.191), confirming that RLP-C levels were stable in menopausal women with DM, indicating consistency with increase of age.

### Effect of RLP-C on the disease progression of menopausal women with DM and CAD

Clinical trials and epidemiological data supported that the effects of estrogen on the cardiovascular system were complex. Studies had shown that rapid, non-nuclear estrogen receptor (ER) signal transduction contributes to transcriptional regulation function of ER mediated the protective effect of estrogen [37]. In the study of the effects of hormone replacement therapy (HRT) on the progression of plasma lipoproteins and CAD in menopausal women, it was found that HRT decreased RLP-C, but increased HDL-C levels, and accelerated the progress of coronary atherosclerosis in DM women [38]. Interestingly, some studies had found that although menopausal women had higher RLP-C levels, they were not affected by HRT and were not associated with the progress of angiographic progression or clinical outcomes of CAD [39]. At the same time, studies had found that although RLP-C decreased significantly after HRT treatment, the process of coronary atherosclerosis in menopausal women also accelerated with the decline of RLP-C [40]. Among the Chinese menopausal women in our study, few patients received HRT treatment, but DM patients accounted for more than 30% of the menopausal women. At present, the relationship between DM and estrogen level was not clear, and the specific effect of HRT therapy in menopausal women with DM and CAD was worthy of further study. At the same time, we speculated that RLP-C was closely related to DM status and the special body state of menopausal women, including hormone levels and unique metabolic status.

## Conclusions

Based on the results of cross-sectional studies of menopausal women, RLP-C could be an independent risk factor for menopausal women with DM and CAD.

### Limitations

(a) This was a cross-sectional study and it lacked basic research as a basis, and in the clinical research of lipids, the measurement methods of VLDL-C and IDL-C were more accurate, and the calculation method of RLP-C might bring a certain degree of errors. (b) A number of cardiovascular risk factor such as lipoprotein A or apolipoprotein B were non evaluated in this study. (c) The study population lacked estrogen data and could not carry out statistical analysis of estrogen in menopausal women population. (d) The enrolled population was all female patients over 50 years old at Anzhen Hospital in 2015, which was relatively limited in patient selection time.

## Data Availability

The datasets used and/or analyzed during the current study will be available from the corresponding author on reasonable requests.

## Abbreviations

RLP-C: Remnant-like particle cholesterol
CAD: Coronary artery disease
DM: Diabetes mellitus
LDL-C: Low-density lipoprotein cholesterol
SBP: Systolic blood pressure
DBP: Diastolic blood pressure
BMI: Body mass index
CVD: Cardiovascular disease
TC: Total cholesterol
TG: Triglyceride
HDL-C: High-density lipoprotein cholesterol
HDL: High-density lipoprotein
LDL: Low-density lipoprotein
RLP: Remnant-like particle
VLDL-C: Very-low-density lipoprotein cholesterol
IDL-C: Intermediate-density lipoprotein cholesterol
LOX-1: Low-density lipoprotein receptor-1
ACS: Acute coronary syndrome
ER: Estrogen receptor
HRT: Hormone replacement therapy
